# Developing family rooms in mental health inpatient units: an exploratory descriptive study

**DOI:** 10.1186/s12913-015-0914-0

**Published:** 2015-06-19

**Authors:** Sophie Isobel, Kim Foster, Clair Edwards

**Affiliations:** Nursing Research, Sydney Local Health District, Concord Centre for Mental Health, Hospital Road, Concord, 2139 Australia; Faculty of Health, University of Canberra, Canberra, Australia; Mental Health Services, Sydney Local Health District, Concord Centre for Mental Health, Hospital Road, Concord, NSW 2139 Australia

**Keywords:** Parental mental illness, Children, Therapeutic space, Family rooms, Family focused care

## Abstract

**Background:**

Family-friendly spaces for children and families to visit inpatient mental health units are recommended in international mental health guidelines as one way to provide service delivery that is responsive to the needs of parent-consumers and families. There is a lack of evidence on the implementation of family-friendly spaces or Family Rooms. This study aimed to explore the development, role, and function of Family Rooms in four mental health inpatient units in a local health district in NSW Australia.

**Methods:**

An exploratory descriptive inductive-deductive design using multiple data sources was employed. Methods included Family Room usage and parental status data over a 12 week period, an open-ended questionnaire, and semi-structured interviews with 20 nurses.

**Results:**

Available parental status data indicated that between 8–14 % of inpatients were parents of dependent children under 18. Family Room usage was multipurpose and used specifically for children & families 29 % of the time. As spaces in the units, Family Rooms were perceived as acknowledging of the importance of family, and providing comfortable, secure spaces for parent-consumers and their children and family to maintain connections. Units did not have local policies or guidelines on the development, maintenance, and/or use of the rooms.

**Conclusions:**

Despite long-standing recognition of the need to identify consumers’ parental status, there remains a lack of systematic processes for identifying parents in mental health inpatient services nationally. Family Rooms as spaces within inpatient units acknowledge the importance of families and are a step towards provision of family-focused mental health care. Recommendations for establishing and maintaining Family Rooms are outlined.

## Background

International mental health guidelines recommend that adult mental health units have an allocated space for families, particularly those with children, to visit consumers while they are hospitalized [1, 2]. Research indicates general consensus from consumers, carers and health professionals that children of parents with mental illness (COPMI) should be able to visit their parents during periods of hospitalization [3] for the benefit of both children and parents. However consumers and staff identify the lack of, and need for, family-friendly spaces so parents can spend personal time with their children [4]. In New South Wales, Australia, national policy has informed the development of a five year plan for mental health services to better address the needs of adult mental health consumers who are parents [5]. This policy framework asserts that family friendly spaces or designated rooms should be established in mental health settings as one way to facilitate family-focused care and provide service delivery that is responsive to the needs of parent-consumers.

For the purposes of this study, Family Rooms are defined as identified spaces fitted out with child-friendly furniture, toys and resources, for children and families to use while visiting mental health settings. There is a lack of evidence on the policy implementation of family-friendly spaces into practice. This paper reports findings from an exploratory study investigating the development, role and function of Family Rooms in mental health inpatient settings.

As mental health services declare their alignment to recovery-focused person centred care [6], there is a need to address philosophical and environmental barriers to delivering such services. While recovery-oriented practice recognises and embraces the possibilities for recovery created by the strengths and capacity of people experiencing mental health issues and maximises self-determination of health and wellbeing [7], admission to mental health units inherently removes people from their daily context. In many units, restrictive mobile phone policies and internet firewalls limit consumers’ interactions; visiting guidelines impact on their connections with family and friends; and locked doors keep people both in and out. When the person at the centre of care is a parent, recognition of their parental role is at risk of becoming subordinate to their individual treatment plan.

The needs of parent-consumers have been recognised through calls for mental health care that is inclusive of children and families [8]. Family-focused care is widely acknowledged as a crucial aspect of effective mental health care delivery [9] and is an approach that respects the uniqueness of consumers and family and aims to provide support to families in their caregiving roles [10, 11]. Family inclusion in care can be seen as a necessary progression from the individualism of person-centred care, but integrating individuals, families and treatment remains a challenging process of policy implementation. One concrete way for Mental Health services to move beyond rhetoric and support individual and family recovery, is to overtly demonstrate a commitment to individuals and their contexts, including designating space for families to visit. Available space, however, is at a premium in established Mental Health inpatient units [12] as rooms are routinely required for interviews, medical reviews, therapies, offices, seclusion or therapeutic purposes such as sensory rooms [13].

The politics of allocating space within units reflects service dynamics and priorities but the philosophy of space and its use links back to debates about the meaning of space itself. Considering the meaning of space is relevant to understanding the implications of space allocation in established mental health units. If space is an absolute as proposed by Isaac Newton in early modern literature [14], and something that therefore exists permanently and independently of matter within or around it, then the importance of a designated space for families lies within its allocation and segregation.

Yet if space is less of a continuous absolute in its own right and rather an intrinsic feature of its properties, or a system of holding or perceiving relationships between things of substance as described by Leibniz in 1716 [15], then allocated rooms for families become meaningless without the presence of the families within them (and the service structures that support them to get there). More recently Williams et al. [16] replicated a similar understanding in determining that hospital environments become therapeutic only when they contribute to a perception of emotional comfort in patients.

If space were to be accepted as an intuition of human faculties of sensibility, or a systematic framework to organise experiences as described by Kant in 1781[17], then perhaps in mental health services, setting aside space for families organises the presence of children and family into the structure of care with an intention that there is fluidity of the space to exist beyond its allocated walls. This is consistent with Douglas & Douglas’ [18] finding that ‘patient friendly environments’ are determined by how people feel and what emotions space creates rather than the spaces themselves. However space is viewed and understood; it is almost surely not of therapeutic benefit on its own [19], without being accompanied by human actions. What has not yet been determined is whether by allocating space for families, families become more or less visible within mental health services. Policy guidelines may recommend the allocation of family spaces but attention also needs to be paid to the relationships between staff, consumers and families within these spaces to justify the intentions of the guidelines and to make the space beneficial. Family-friendly spaces can facilitate opportunities for positive interactions between hospitalized parent-consumers and their children, and between staff and families, while also allowing staff opportunities to observe parents’ and families’ contact [20]. This observation is not purely for supervisory or protective purposes, but is important for supporting and enhancing familial relationships, for acknowledging consumer parenting roles and identities, and for demonstrating the value staff place on these roles [21].

Nurses play a central role in supporting children and family visiting inpatient mental health settings. Literature on nurse – family interactions indicates that while nurses are generally supportive of children and family visiting, they lack clarity and confidence in their role in facilitating visits, and acknowledge a lack of policy and guidelines to support visitations [22, 23] Further, there has been limited exploration of the environments in which nurse-family interactions occur, and little reference to the potential therapeutic benefits of space for families in these settings (Cleary et al. paper; Korhonen et al. 2010). Despite policy and framework guidelines, Family Rooms are not routinely allocated in inpatient mental health units in Australia and there is a lack of published literature on their establishment.

Establishing Family Rooms is one of the first steps towards family-focused care in mental health services. With little published literature on how, by whom, and when family rooms are being used in these settings, there is a clear gap in knowledge on how this policy initiative has been translated into practice. This study provides valuable new evidence on Family Rooms in mental health settings in Australia, with implications for the establishment of Family Rooms in other settings and contexts.

The study examined the establishment and use of Family Rooms in Mental Heath Inpatient units. The aim of the present study was to explore the development, role, and function of Family Rooms in four mental health inpatient units in one local health district in NSW Australia. The four units comprised three acute adult admission wards and one rehabilitation unit (Table 1).

**Table 1:** Mental health inpatient units characteristics

Acute admission 1	Acute admission 2	Acute admission 3	Acute admission 4
• Consumers within first two years of diagnosis	• Consumers linked with service >2 years	• Consumers with acute episode or relapse of mental illness or disorder	• Consumers with sustained mental illness requiring psychosocial intervention and support
• 24 beds	• 24 beds	• 20 beds	• 20 rehabilitation/ recovery beds
• 12 bed female High Dependency Unit (HDU) attached	• Average length of Stay 30 days	• 10 HDU beds	• 10 Long stay beds
• Average Length of Stay 16 days (8 days HDU)		• Average Length of Stay 22 days	• 5 forensic beds
			• Average Length of Stay 101 days

## Methods

As little is known about the establishment and use of Family Rooms, an exploratory descriptive design [24] with multiple data sources was used. A project advisory group guided the study, comprising consumer, carer, and senior nurse representatives from the Local Health District. Due to a lack of systematic documentation of parental status in mental health settings, data were individually gathered from each unit on the number of parent consumers over a 12 week period. In order to gain an understanding of the use of Family Rooms, descriptive data were gathered via a written log maintained for up to 12 weeks in each room and filled in by staff when the room was used. Log data included when, by whom, for what purpose and for how long the Family Room was used. To ascertain the development and structure of Family Rooms in the four units, a brief, purposefully designed, questionnaire was completed by Nurse Unit Managers, with 7 open-ended questions asking about the process of and impetus for, room development, any obstacles to development, decision making processes and descriptions of the unit, the room, and its use. Paper questionnaires were distributed to Nurse Unit Managers with information about the study and returned via internal mail to the Primary Investigator. All distributed questionnaires were returned.

Finally, an in-depth semi-structured face-to-face interview [25] of up to 60 min was conducted with a purposive sample of 20 nursing staff from across the four units. Due to the exploratory nature of the study, and the lack of prior research, interviews explored what had occurred in relation to child/parent visits prior to the room’s development, how the rooms were developed, the structure, function and use of the rooms, and nurses’ understanding of the room’s purpose. Questions also explored what was beneficial in using the rooms, and how room usage could be improved or modified.

The study received ethical clearance from the Sydney Local Health District Ethics Committee and the University of Sydney. Nursing staff in the four inpatient units received verbal and written information on the study. The first author informed staff about the study at team meetings and handovers and invited staff to participate. Inclusion criteria were registered nurses from a range of seniority and experience levels, who had experience in using the Family room in the unit. Twenty nurses (five from each unit) who provided written informed consent participated in the interviews. Interviews occurred in 2013, and were conducted in a quiet room in the health service at a mutually convenient time, lasted up to 60 min, and were digitally recorded.

An inductive-deductive analytical approach was used. Data on parent status and log data on room usage were entered into Excel and reported using frequencies and percentages. Open-ended questionnaire data were entered into Excel, and interview transcripts were transcribed verbatim. Interview and questionnaire data were managed using NVIVO 9 software. Conventional content analysis was used for integrated questionnaire and interview data. This inductive analytic approach is appropriate for studies describing a phenomenon [26]. All data were read for key concepts and coded. Codes were sorted and clustered into emergent categories by the first investigator. In an iterative process, emergent categories were then discussed and refined by the research team until agreement was reached on the final categories.

## Results

### Parental status

From the data gathered, inpatients identified as the biological parent of a child under 18 years either in or out of their guardianship ranged between 8 % and 14 % across units over 12 weeks. (Total number of parents *n* = 0−12, av. 3.5, out of total inpatients per unit, *n* = 24−38, av. 31, combined as a percentage over 12 week data period).

### Family room usage

Summary findings from logs, which were not consistently filled in by staff over the 12 weeks, indicated that children in the units ranged in age from 0 to 16 years and family groupings ranged from one to up to eight children visiting their parent at one time. Children were primarily brought in by family members as well as escorts, including guardians or child protection workers. A total of 89 h of room usage across the four units was recorded. Rooms were used for a range of purposes, including family visits (Table 2). Visits off hospital grounds were not captured by the log data.

**Table 2:** Family room usage across four units

Room usage purpose	Episodes	Total hours	Percentage of total usage hours
Clinician Led Family meeting (no children)	6	6	7 %
Clinician Led Family meeting (with children)	3	3	3 %
Family Visit (no children)	10	12	14 %
Family Visit (with children)	11	23.5	26 %
Clinical Use (no children)	56	40	45 %
Other use (no children)	7	4.5	5 %
TOTAL	93	89	100

### Participant demographics

Twenty nurses participated in interviews. All were Registered Nurses, six were employed in more senior clinical roles. Ages ranged between 20–59 years and years of experience in mental health ranged between 2 years and 35+ years, with an average of 15. Thirteen had completed or were currently undertaking a postgraduate qualification. Seven participants were male and thirteen were female.

### Questionnaire and interview findings

Four categories were developed from content analysis: Establishing Family Rooms; Aesthetics of Family Rooms; Purposes of Family Rooms; and Challenges of Family Rooms.

#### Establishing family rooms

Nurses spoke about a range of pathways to establishing the rooms, including the impetus for, and process of, room development and what happened prior to its development. All nurses described that prior to having a Family Room, when children visited units they would remain with the person who had escorted them, and generally visited their parent in the unit, foyer, or garden. Nurses were uncertain whether an increase in family and child visits had occurred as a result of having a designated space. Many assumed that the room increased comfort for children and families and therefore might have increased visiting behaviours. One nurse hypothesised:

*“I think, there are a lot of people who, perhaps, would be more likely to bring their children in to visit because there’s a suitable place for them to go, that’s safe” (N4)*

*“Just having a space that’s a bit more inviting to have family over, like I said, it was quite dull and a bit shabby looking before, so having something that looks newer, a bit painted, a bit brighter, a bit more cheerful, a bit more – like I said, inviting, a bit warmer for them to come in”. (N12)*

Nurses also identified that Family Rooms were not standard in mental health units and that the room’s presence in their unit seemed to be determined by a range of factors including the motivation of staff; individuals who drove their development; the characteristics of the patient population; and also luck and timing. Existing policy was not seen to be a determinant in the rooms’ development.

*“We are lucky here…it’s a big ward where we happened to have a room that they could take up as a family room” (N3).*

*“I’m really proud to be on a ward where there are nursing staff who…start this kind of initiative themselves without… “Oh you need to do this”....This was staff who saw a potential and a need and actually took the initiative… and used their own time, … efforts and energy to actually get this going” (N4).*

Family Rooms had been established for between one and five years. Each room was established in different ways, as described in Nurse Unit Manager questionnaires, initiated either by nurses, managers or allied health staff who identified the need for ‘safe and therapeutic’ spaces for families. Rooms were developed using existing funds or through small grants. Decisions about the set up were made internally by staff, and external or managerial support and funding was helpful in developing the room. Obstacles to room development included the slow process of using internal suppliers for goods, and concern from unit staff about the re-allocation of interview room space. All rooms were converted interview spaces and most remained multi-purpose as confirmed in interviews:

*“We use it for visits…assessments....meetings…doctors…supervision…but first and foremost it’s a family room. So if any of that is happening and a family come to the door, people get up and go” (N3)*

*“Doctors use it for interviews.. but it is the room that people know is the family friendly room and when people come with children, that’s where you direct them” (N18)*

#### Aesthetics of family rooms

Despite different pathways of establishment, the rooms had some shared aesthetics. Nurse Unit Managers described that they were decorated in an ad hoc way by motivated staff and maintained in a similar manner; no unit had policies or guidelines on maintenance of the rooms, for example, who cleaned or tidied toys. Some units used funding to purchase toys, furniture and paint, while others relied on contributions from staff. The painting was done by staff, while external sources were required for ordering furniture and custom fixtures.

All rooms were altered to look more familiar and comfortable. New furniture was used to encourage engagement and relaxation, including couches, ottomans and children’s tables and chairs. Rooms were colourful, and often painted with a mural or bright colours. Two of the rooms had a theme for their paintwork, something staff perceived made the space more friendly and appealing. Nurses mentioned that the room was increasingly being identified as relaxing by consumers - even when families were not visiting.

*“I think it’s less of a formal environment is probably one of the most important aspects of it…it gives it a more homely and I suppose less institutional [feel]…people are more likely to become a bit more relaxed” (N6)*

Toys were identified as important in making children feel comfortable and providing stimulation and activity during visits. Toy donations from staff were a regular occurrence but staff were unclear about whether toys needed to be assessed for safety prior to use. One room had a TV and electronic game consoles. Some nurses considered that time in the room should be used to strengthen connections between family rather than playing with games, others considered the TV and games as helpful in relaxing and/or distracting child/ren. Nurses identified that even in the room where TVs and game consoles were provided, they were not observed to be used often by children.

Pamphlets related to support groups, community family support services, parenting and information on mental health were available in all rooms. These were displayed on the walls and families were free to access and use them as they pleased.

#### Purposes of family rooms

Nurses identified a range of purposes and benefits of the rooms for children, parents, families and staff. Many nurses saw the allocation of space for Family Rooms as a symbolic acknowledgment of the importance of family to consumers, with one describing:

*“a family room is basically saying, this [family connection] is so important we’ve made a room specific for that and there is an expectation that families can visit and use this space…it places emphasis on family. By its mere existence it’s pointing to the fact that this is important…so much so that we’re allocating a whole room to it…it’s significant when you’re giving away real estate” (N20)*

Nurses also identified that this acknowledgement needed to be more overt by the rooms being clearly labelled as ‘The Family Room’ so that staff acknowledged and followed its intended purpose:

*“People need to know what they are. They need to be called something. People need to be aware of what they’re used for and how they’re meant to be used and why they were actually put in places [units] in the first place” (N18)*

Nurses saw the room as allowing children to play, reconnect and talk freely with their parent/relative in a comfortable environment. Families sometimes spent extended periods there sharing food and conversation in what was seen as a comfortable and secure environment. Nurses had also observed that parents were often calmer following visits from their family. Maintaining connections with children and families was frequently identified as being valuable for consumers’ recovery.

“*We know it’s a safe room. We know the door is locked. That they’re not going anywhere. We know it’s beneficial in that we can get the patients and children to interact more. They can play together in here” (N14)*

The rooms were perceived by nurses to promote safety through the physical separation of children and family from other consumers, primarily by reducing the risk of children being exposed to possible physical or emotional harm on the units. The rooms were also observed to protect unwell consumers on the unit who could be overwhelmed by the stimuli of young children. Staff reported that rooms established in visible positions on the unit were of additional benefit as they allowed nurses to observe and supervise patients and visitors to minimise the chance of possible distress:

*“sometimes children can be a little bit much for people who are suffering from mental illness…some children can be quite intense” (N6).*

*“the ward, itself, is a very noisy environment…it’s a lot of stimulus, a lot of noise… [the family room] is like a nice little sanctuary” (N4)*

Overall, nurses reported feeling more comfortable when the designated rooms were used for family visits, with one observing: *“I just feel better when they [children and family] are in there” (N4)*

#### Challenges of family rooms

A number of challenges were identified in the use of Family Rooms. These related primarily to staff and unit factors. No challenges were identified for parents, families and children. Nurses described that parent-consumers needed to be appropriately assessed prior to using the room, which could be time-consuming. Assessment referred to mental state, the current relationship with family and children, and any risks to safety for visitors, consumers or staff. If consumers were insufficiently assessed, nurses considered that the safety of children and visitors could theoretically be compromised, so they often spent time supervising visits until safety could be established.

*“When in doubt, you would go to the doctors and say, “This person’s here with this young child. Let’s talk about it together. I haven’t let them see each other yet. Do we think it’s okay for this to occur?”*… *there’s no formal checklist to say whether yes this person’s [parent] safe or no this person’s not safe” (N18).*

Nurses identified that rooms could also potentially be difficult for staff to upkeep. Rooms were sometimes left untidy and equipment went missing. Nurses did not identify additional strain or tasks from this, but rather, posed it as a potential challenge. Further, they considered that the location of the Family Rooms in units was rarely ideal (Table 3).

**Table 3:** Site location of rooms in units

Unit 1	Unit 2	Unit 3	Unit 4
• Within locked unit	• Within locked unit	• Within locked unit	• Within foyer of unlocked unit
• Out of sight of nurses’ station	• Within sight of nurses station	• Opposite nurses’ station	• Within sight of nurse unit manager
	• Access requires walking through unit.		
	• Alternate room often used outside of locked unit.		

Rooms located just outside the units were viewed favourably for safety and ease of access but posed logistical challenges. Nurses recommended that rooms be either outside the unit entrance or close to the nurses’ station for reasons of observation, safety and comfort of families. Family Rooms that involved walking children and families through the unit were seen as undesirable due to potential for distress for children and family members. One unit continued to use an existing visitor’s room over the newly fitted out Family Room, due to concerns about the new room’s location being further from access points of unit and requiring children to be walked through the unit to enter. This was not a decision discussed openly but had developed through a culture of practise.

*“It’s not well located. The idea is great....it looks good…the furniture is appropriate…there’s toys for the kids, but probably not a good spot for it” (N2)*

Nurses reported that when children did not visit their parents, it was usually at the parent-consumer’s request. Nurses hypothesised that parents could have concerns about stigma and fears about their children being exposed to a negative environment and therefore refused to have their children visit. Nurses also identified that it was easy to become desensitized to the experience of entering a mental health facility as a visitor and as such the unit environment may not offer the level of support and encouragement that families required:

*“Stigma still plays a large role, people don’t understand mental illness, they’re frightened of it…they’re frightened of the types of people that might be in an inpatient service....It’s almost to the degree you’d think it was contagious” (N20).*

*“I think that some people who work here....probably underestimate the impact that coming into a mental health setting can have on some people who aren’t used to it.” (N6)*

Typically, in all units, nurses escorted children and visitors from the main entrance to the Room. Nurses reported that most units required visitors to phone in advance to check the consumer’s suitability for visitors and whether the room was available. However, in their experience most visitors arrived unannounced.

## Discussion

Despite international literature recommending that adult mental health units have an allocated space for children and families to visit [27–29] there has been little or no discussion of how these spaces function logistically, what structures are needed to support their existence, and how they may fit within existing services [30, 31]. This study provides new information and considerations for developing and using Family Rooms in a range of inpatient contexts.

Parental status statistics for the 12 week study period were not able to be retrieved with accuracy from the existing documentation processes and instead required individual support from staff. Systematic identification of children has long been recognised as a necessary first step towards family-focused care [32, 33] and while there is documentation available to capture parental status in the relevant health district, this information is not systematically recorded. The challenges faced in retrieving parent status in this study highlights the lack of progress in systematic identification of parents in mental health settings. The 8 %–14 % of identified parents of children under 18 across the units is lower than previously published data that estimates 20–30 % of all mental health service users are parents [34, 35]. The lower incidence reflects the inpatient-only focus of this study, the admissions during the study period, as well as the potential for under-reporting in data collection reliant upon individuals.

There was wide variation in the frequency of children visiting units with no apparent demographic determinants of this difference at a unit level. Room usage logs were notably inconsistent in their completion and provided limited insight into the demographic relationship between parent-consumers and children & family visits. Nevertheless, the available data indicated that nearly a third (29 %) of room usage was for children and families, a further 21 % for adult family members, with the rest (50 %) for other clinical/non-clinical purposes which included consumer interviews, consumer assessments, staff supervision sessions, medical interventions and consumer time-out. In the context of stretched space allocation, this indicates the reality of multi-purpose spaces that prioritise family usage but do not preclude other uses. Room usage also suggests that while the rooms were being used for their primary purpose, there is potential for usage ‘creep’ over time and a concomitant need to prioritise and safeguard room usage for Family purposes. Staff role/s may need to include a ‘champion’ for room usage and to ensure maintenance of the room’s purpose over time.

Hospital environments and their organisational cultures are known to include a coalition of values and support behaviours that reflect cultural norms across wards [36]. Within this understanding, a change in environment (i.e. establishment of a Family Room) may also represent a change in values and behaviours towards family focused care. This is consistent with previous findings [37] that clinicians in acute mental health care operate from a framework that is in part created by the space they occupy and that altering environments can have a profound impact on human relationships. Research addressing the impact of space upon mental health outcomes highlights the importance of actively creating therapeutic milieu within spaces [38].

In linking Family Rooms back to the philosophical meaning of space itself, allocating the space is itself of impact and significance, fitting it out with colours, furnishings and resources is beneficial, but it is staff facilitation of family experiences within the space that will impact mental health care. Space on its own is not neutral [39] and is imbued with meaning even when empty [40]. While a change of environment may influence the culture of the unit, a more explicit articulation of staff roles, values and expectations in regard to the space is also required to support this change. As, Stickley and Freshwater [41] note, stopping to appreciate the qualities and meanings of space itself may be dismissed as a conceptual process of a nebulous state; yet consideration of the philosophical underpinnings of the meaning of space is helpful in considering the meaning of spaces such as Family Rooms within the wider health care environment and for understanding the importance of the substance and actions that fill them.

Findings from this study indicate that designated Family Rooms can be seen to contribute to the therapeutic landscape of mental health inpatient units. Having such spaces indicates respect for consumers’ need for privacy and social relationships [42], acknowledges their parental status and need for connection with children and families [43] and supports the process of individual and family recovery in mental health. The existence of Family Rooms, processes to support parents in their parental role and potential for positive inpatient experiences with family, may also help alleviate self-stigma for consumers [44]. Family rooms are a metaphorical ‘welcome mat’ for children and families into potentially unwelcoming environments. Parent-consumers and family carers have previously acknowledged that visiting in the open unit is, at times, not safe or appropriate [45]. With broader recognition that mental health units are conflictingly unsafe-safe environments [46], Family Rooms may need to be considered a necessity not a luxury.

The development of Family Rooms by the health service in this study can be understood as a pioneering act of translation of mental health policy and a move towards family-focused mental health practice. The use of Family Rooms and provision of family support constitutes ‘good mental health nursing practice’ [47] and sits within of a framework for family-focused mental health nursing practice that addresses the wellbeing and resilience of children, parents and families [48]. Evidence indicates that preventive interventions for children whose parents have mental illness can reduce the intergenerational risk of developing mental illness by up to 40 % [49]. There is untapped potential for family room spaces to be used therapeutically by staff through psycho-education and brief interventions to support parenting and family.

Our findings indicate there is an urgent need for implementation of accurate and timely processes for identifying parent status in mental health services. This study highlights that current systems of documenting parental status are inconsistently completed. This has significant implications for care provision. Accurate assessment and documentation of parental status of consumers is needed upon and throughout, contact with services in order to provide family-focused care which addresses their parenting role and the needs of their children and family.

The shift towards family focused care inevitably involves small acts of progression in practice as well as larger shifts in policy and service structure. Despite a significant period of the existence of policy and guidelines, our findings indicate that Family Room development appears to occur in the context of motivated individual staff who perceive a need and enact upon it, with or without support.

This was an exploratory study in a metropolitan area of a major city, and the findings and recommendations may not be applicable to all settings or regions. The study is limited by lack of complete data on room usage, and lack of audit data on parental status documentation. There is a need for further investigation into the development and use of Family Rooms in a wider range of inpatient settings; the experiences of children and family who visit in the rooms; and the role of nurses and other health professionals in working with families in the context of Family Rooms.

## Conclusion

This study found that Family Rooms are multi-purpose space re-allocations that can be implemented in mental health inpatient units with relatively few additional resources and can be seen to provide a concrete foundation for, and commitment to, providing family-focused care. Although policy exists on Family Rooms, this is not necessarily translated into systematic establishment of rooms. There is a need for guidelines on the structure, purpose/s and use of Family Rooms to make most effective use of rooms and to support the consumers, families and staff who use them. Recommendations for establishing Family rooms are outlined (Table 4).

**Table 4:** Key recommendations for establishing family rooms

Location of rooms:	– label the room ‘Family Room’
	– close to nursing station
	– high visibility of room within unit
	– ease of access to unit entry
	– consider level of passing traffic and noise
Aesthetics & content:	– use bright colours on walls
	– include appropriate toys/activities for varying age ranges
	– include information and brochures on parenting & support services
	– decide whether to include electronic equipment/games
	– use comfortable & child-friendly furniture
Policy and guidelines for use of rooms:	Develop written policy for use of room, including role/s and processes for:
	– scope of room purpose/s and usage
	– staff participation & role in child & family visits
	– provision of psycho-education & support to parent, children & family
	– assessment of parent wellbeing prior to visits
	– supervised versus unsupervised family visits
	– process for escorting families to and from room
	– room entry & exit points (health & safety requirements)
	– cleaning & maintenance of toys and contents
	– nominate a clinical leader or champion for Family room
